# Supplementary material for: Identification of photoperiod-induced specific miRNAs in the adrenal glands of Sunite sheep (Ovis aries)
Source: Front Vet Sci. 2022 Jul 22;9:888207. doi: 10.3389/fvets.2022.888207 (PMC9354845; doi:10.3389/fvets.2022.888207)
Supplement: Supplementary file 1 [file Data_Sheet_1.ZIP › Supplementary file/miRDeep2 file.zip!/miRDeep2 file/Novel_Structure/LP42A2/Novel_323.pdf]

Provisional ID	:	NC_019471.2_24171
Score total	:	1.8
Score for star read(s)	:	-1.3
Score for read counts	:	0
Score for mfe	:	1.5
Score for randfold	:	1.6
Score for cons. seed	:	
Total read count	:	57
Mature read count	:	57
Loop read count	:	0
Star read count	:	0

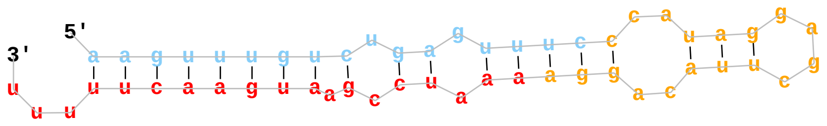

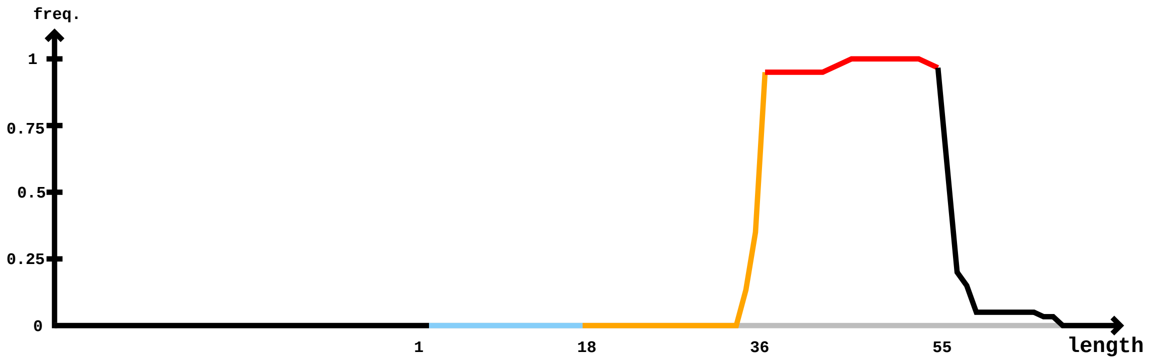


```
                                        Star                                  Mature
5'- cuuuuaaauuuaugcacauauguauuggguuggccaaaaguuugucugaguuucccauaggagcuuacaggaaaauccgaaugaacuuuuuggccaacccauauauugg   -3'    exp
    ................((.((((((.((((((((((((((((((((.((.(((((..(((....)))..))))).)).).)))))).))))))))))))))))))))).   reads     mm        sample
    .......................................................................gaaaauccgaaugaacuuu...................   1         0         seq
    .......................................................................gaaaaCccgaaugaacuuuu..................   1         1         seq
    .......................................................................gaaaauccgaaugaacuuuuu.................   2         0         seq
    .......................................................................gaaaaucUgaaugaacuuuuug................   1         1         seq
    .......................................................................gaaaauccgaaugaacuuuuug................   3         0         seq
    ........................................................................aaaauccgaaugaacuuuuu.................   8         0         seq
    ........................................................................aaaaCccgaaugaacuuuuu.................   1         1         seq
    ........................................................................aaaaucAgaaugaacuuuuug................   1         1         seq
    ........................................................................aaaauccgaaugaacuuuuugg...............   1         0         seq
    ........................................................................aaaaCccgaaugaacuuuuugg...............   2         1         seq
    .........................................................................aaaCccgaaugaacuuuuu.................   28        1         seq
    .........................................................................aaauccgaaugaacuuuuu.................   1         0         seq
    .........................................................................aaauccgaaugaacuuuuug................   1         0         seq
    .........................................................................aaaCccgaaugaacuuuuuggc..............   6         1         seq
    ...............................................................................aaugaacuuuuuggccaaccca........   1         0         seq
    ................................................................................augaacuuuuuggccaacccaGa......   1         1         seq
    .................................................................................ugaacuuuuuggccaacccaua......   1         0         seq
```
